# [Retracted] Pro-metastatic intracellular signaling of the elaidic *trans* fatty acid

**DOI:** 10.3892/ijo.2026.5935

**Published:** 2026-09-02

**Authors:** Kiyomu Fujii, Yi Luo, Rina Fujiwara-Tani, Shingo Kishi, Song He, Shuyun Yang, Takamitsu Sasaki, Hitoshi Ohmori, Hiroki Kuniyasu

Int J Oncol 50: 85-92, 2017; DOI: 10.3892/ijo.2016.3797

Following the publication of the above paper, an interested reader has drawn the Editor's attention to the fact that the tubulin control blots featured in Fig. 3 on p. 88 were strikingly similar to the control blots subsequently featured in a figure in another paper that was published by the same research group in the journal *Oncotarget*, where the experimental conditions were reported to be different. A subsequent investigation of the data in this paper undertaken by the Editorial Office revealed that various of the western blots included in Figs. 2A and B, 3E and F, and 4C and D appeared to have been duplicated within and/or between these figure parts; moreover, various of the data in these figure parts were remarkably similar to data which appeared (either previously or subsequently relative to the publication date of this article) in other papers written by different authors at different research institutes.

Given that the contentious data mentioned above have apparently re-appeared in a range of other publications, the Editor of *International Journal of Oncology* has decided that this paper should be retracted from the Journal. The authors were asked for an explanation to account for these concerns, but the Editorial Office did not receive a reply. The Editor apologizes to the readership for any inconvenience caused.

